# Measurement of δ^1^-Pyrroline-5-Carboxylic Acid in Plant Extracts for Physiological and Biochemical Studies

**DOI:** 10.3390/metabo15120777

**Published:** 2025-12-02

**Authors:** Giuseppe Forlani, Flavia Trupia

**Affiliations:** Department of Life Science and Biotechnology, University of Ferrara, Via L. Borsari 46, I-44121 Ferrara, Italy

**Keywords:** δ^1^-Pyrroline-5-carboxylic acid (P5C), proline–P5C cycle, colorimetric assay, enzymatic assay, proline dehydrogenase, P5C dehydrogenase

## Abstract

Background/Objectives: δ^1^-Pyrroline-5-carboxylic acid (P5C) is a key intermediate in both the pathways leading in plants to proline synthesis, as well as in the proline catabolic route that takes place in the mitochondrion. Instead of being further oxidized, the P5C released in the latter process could be transferred to the cytosol and reduced back to proline in an apparently futile proline–P5C cycle, which seems to play a role in the plant response to pathogen attack. To date, studies on this cycle and the enzymes involved have been hampered by the lack of an efficient protocol to measure P5C in plant extracts. Methods: The experimental conditions allowing P5C to be extracted from plant tissues, stabilized, concentrated by cation-exchange chromatography, and quantified by reaction with *o*-aminobenzaldehyde were set up and validated. Results: The optimized protocol was shown to be capable of detecting P5C accumulation in tissues of a proline-treated *Arabidopsis thaliana* p5cdh knock-out mutant, as well as allowing the measurement of proline dehydrogenase activity in partially purified extracts from plant cultured cells. Conclusions: The method herein described overcomes current limitations in detecting and quantifying P5C and will facilitate the study of the related enzymes and the elucidation of its debated role in plant metabolism.

## 1. Introduction

**Figure 1:**
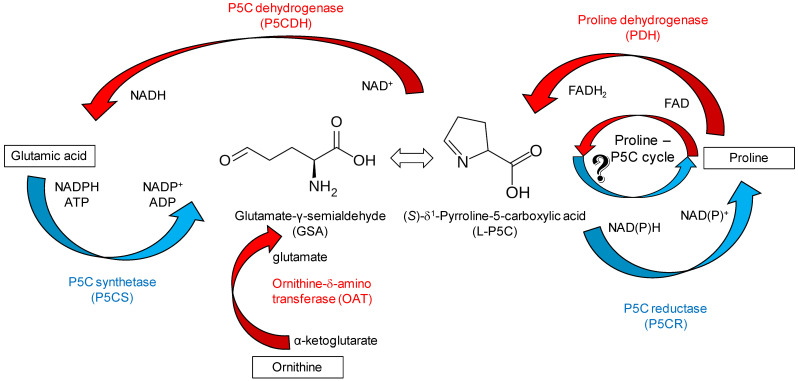
Metabolic relationships of l-P5C. The intermediate is derived from the spontaneous cyclization of GSA, which is produced in both the glutamate and the ornithine pathways leading to proline biosynthesis. l-P5C is also produced during proline oxidation in the mitochondrion. A proline–P5C cycle has also been hypothesized, but the occurrence of a transporter for P5C from the mitochondrion to the cytosol has not been demonstrated, yet. Reactions that take place in the cytosol are emphasized in blue; those in the mitochondrion are shown in red.

(*S*)-δ^1^-Pyrroline-5-carboxylic acid (l-P5C, 3,4-dihydro-2H-pyrrole-2-carboxylic acid) is a central intermediate in the metabolism of the amino acids of the glutamate family in plants (Figure 1) [1,2]. During proline biosynthesis, a bifunctional, cytosol-localized P5C synthetase (P5CS) reduces glutamic acid to glutamate γ-semialdehyde (GSA), which in solution spontaneously undergoes dehydration and cyclization, yielding l-P5C [3,4]. GSA is also formed in the mitochondrion by the activity of ornithine-δ-amino transferase (OAT) [5]. In both cases, l-P5C is then reduced in the cytosol to proline by P5C reductase (P5CR), which uses either NADH or NADPH as the electron donor [6]. Excess proline can be oxidized back to glutamate in the mitochondrion, where a membrane-bound flavoenzyme, proline dehydrogenase (ProDH), transfers reducing equivalents directly to the respiratory chain, with the release of l-P5C [7]. The latter is oxidized in turn to glutamate by a P5C dehydrogenase (P5CDH), with concomitant production of NADH [8]. Under certain conditions, a metabolic shortcut seems to take place in which the l-P5C formed by ProDH, instead of being further reduced to glutamate, is transferred to the cytosol and reduced back to proline by P5CR [9]. This apparently futile proline–P5C cycle has been hypothesized to transfer reducing equivalents from the cytosol to the mitochondrion [10], but its occurrence is far from being conclusively proved [11].

The physiological role of proline metabolism in plants goes well beyond fulfilling the need of building bricks for proteins. Over recent decades, increasing evidence has emerged supporting a main role for proline biosynthesis in the plant response to abiotic stress conditions, when proline accumulates to ensure osmotic compensation and protect proteins and membranes against denaturation [12,13,14]. Conversely, proline catabolism seems to be involved in the reaction of higher plants to pathogen attack, being associated with the induction of programmed cell death (PCD) [15,16]. Such a strikingly different outcome of proline metabolism most likely depends on how l-P5C is metabolized [11]. During incompatible pathogen infections, high expression of P5CS and ProDH in connection with low levels of P5CDH is expected to increase l-P5C concentration, which somehow triggers PCD [17]. Consistently, the induction of P5CDH by fungal effectors has been described in rust–flax compatible interactions [18]. The mechanisms underlying such effects are still unclear, since l-P5C could induce apoptosis per se or through generation of reactive oxygen species (ROS). On the other hand, proline synthesis plays a role in maintaining a proper NADP^+^/NADPH ratio [19] and may serve as an overflow mechanism to dissipate excess reductants under conditions in which photosynthesis is impaired [20].

In many bacteria, ProDH and P5CDH activities are present in a single, bifunctional proline oxidase [21]. The presence in plants of two separate enzymes, despite the consequent risk of l-P5C accumulation leading to cytotoxic effects, has been invoked as evidence supporting the occurrence of the proline–P5C cycle. This metabolic shortcut has been quite unambiguously demonstrated in mammals, where it functions to amplify proline-dependent ROS production [22,23,24]. However, in mammals, two P5CR isoforms are present in the mitochondrion [25], whereas in plants, P5CR seems exclusively localized in the cytosol [26]. As a consequence, l-P5C needs to be transported through the mitochondrial membranes, but to date, experimental evidence for a specific l-P5C transporter is still lacking. Moreover, some recent results showed, in *Arabidopsis thaliana*, physical interactions between ProDH and P5CDH, as well as between ProDH and OAT [27]. Co-migration experiments suggested the formation in the mitochondrion of a protein complex that could facilitate substrate channelling. If so, l-P5C would be sequestered and could not be transported into the cytosol, making the proline–P5C cycle unlikely. This notwithstanding, it cannot be excluded that these supramolecular complexes may assemble or disassemble according to different environmental conditions, allowing l-P5C to be accumulated to promote PCD or to be transported into the cytosol to fuel the proline–P5C cycle. Overall, despite the plethora of studies and these recent new findings, the regulative switches and the molecular mechanisms by which proline metabolism can ensure adaptation or defence through such strikingly different outcomes are far from being fully understood.

A drawback hampering a deeper comprehension of several aspects of proline metabolism is represented by the properties of l-P5C itself. This compound is highly unstable in solution at a neutral pH [28] and is not commercially available from reliable sources. Its steady-state intracellular concentration is maintained at very low levels (<0.02 μmol [g fresh weight]^−1^) [29]. As a consequence, detection and quantitation of P5C by HPLC is not straightforward. Pre-column derivatization with *o*-phthaldialdehyde, the most commonly used method for amino acid analysis, does not work with secondary amino acids [30]. Other procedures can be used, such as the phenylisothiocyanate [31] and the dabsyl chloride [32] methods. However, due to the presence in cell extracts of free amino acids at levels at least two orders of magnitude higher, P5C detection with such methods would require preliminary steps to concentrate and at least partially resolve the intermediate from other reactive substances. Recently, HPLC-MS analysis that combines the separation power of HPLC with the sensitive detection and mass analysis of MS has been described as being able to quantitate P5C in biological samples. 5-(Diisopropylamino) amylamine derivatization coupled with UHPLC-Q-TOF/MS was used to profile carboxylic acids in plasma samples, leading to the identification of P5C as a biomarker of depression [33]. A UHPLC-Q-TOF (Quadrupole Time-of-Flight) mass spectrometric analysis was described to detect P5C and its spontaneous reaction products with pyridoxal 5′-phosphate, malonic acid, and acetoacetic acid in hyperprolinemia type II patients [34]. However, such approaches require expensive equipment, trained personnel, and complex data analysis and are not always affordable. The availability of a simple protocol based on LC and spectrophotometric analysis would greatly facilitate P5C quantitation and help in elucidating its role in plants.

Two colorimetric assays have been described to detect and quantify l-P5C, namely, a specific reaction of ninhydrin under strongly acidic conditions that produces a cherrish red adduct with a peak of absorbance at 535–540 nm [35] and a reaction with *o*-aminobenzaldehyde (*o*AB) that yields a yellow product with maximal absorbance at 444 nm [28]. However, both methods suffer from interference by other compounds commonly present inside the cell. Though to a lesser extent, acid ninhydrin reacts with most free amino acids, *o*AB, besides reacting with other biological semialdehydes, has to be dissolved in ethanol, which causes protein precipitation in crude extracts. Therefore, such assays can be used to quantify l-P5C only in purified preparations. Consistently, the enzymes that produce or utilize l-P5C are assayed by measuring the concomitant reduction/oxidation of NAD(P)^+^/NAD(P)H [36,37] or, as for ProDH, the reduction in artificial electron acceptors [7].

In this work, we tried to set up a protocol for a preliminary enrichment step based on liquid chromatography that would support the use of one of the above-mentioned colorimetric assays to detect and quantify P5C in crude plant extracts. The removal of interfering materials and a concomitant enrichment of the compound could allow the measurement of the lowest P5C levels that are present in plants tissues without the need for complex and expensive equipment, such as that required for HPLC-MS analysis, making the study affordable in most laboratories. Similarly, interference removal could also be used to quantify P5C in assay mixtures, representing an alternative to current assay methods for measuring the activity of P5C-metabolizing enzymes.

## 2. Materials and Methods

### 2.1. Reagents

Unless otherwise specified, the chemicals used were obtained from Sigma-Aldrich-Supelco-Merck Life Science (Milan, Italy) and were of analytical grade.

### 2.2. Synthesis and Purification of dl-δ^1^-Pyrroline-5-carboxylic Acid

dl-P5C was synthesized by the metaperiodate oxidation of hydroxylysine according to Williams and Frank [28]. About 600 mg of dl-5-hydroxylysine hydrochloride (3 mmol; Sigma H0377) was weighed under dim light and dissolved into 35 mL of double-distilled water in a brown glass bottle; 644 mg (3 mmol) of Na metaperiodate (Sigma S1878) were similarly dissolved into 50 mL of water, and the pH was brought to 7.0 with 2 M NaOH. Both solutions were equilibrated on ice and then mixed rapidly. The oxidation of hydroxylysine was allowed to proceed for 8 min, after which the excess metaperiodate was destroyed by the addition of 950 μL of 1 M glycerol (Sigma G7893). After a further 2 min, the reaction was blocked by the addition of 2.46 mL of 2 M HCl (Carlo Erba Reagents 404067000, Milan, Italy). The reaction mixture was brought to 100 mL with water and immediately loaded onto a Dowex AG50 WX4 (200-400 mesh) (Supelco 44473) column (acid form; 5 cm diameter, 50 mL bed volume) equilibrated with water, at a constant flow of 90 mL h^−1^. After washing with 50 mL of 50 mM HCl, isocratic elution proceeded with 1 M HCl (Carlo Erba Reagents 404097000), while collecting 6 mL fractions. Fractions containing P5C were pooled, filter-sterilized (0.45 μm), and stored in the dark at 4 °C.

### 2.3. Quantitative Determination of δ^1^-Pyrroline-5-carboxylic Acid

P5C was quantified by either the acid ninhydrin [35] or the *o*-aminobenzaldehyde (*o*AB) [28] method, with minor modifications. In the former case, 15 μL of proper dilutions of the sample was mixed with the same volume of a 3 M Na acetate (Sigma S2889) solution in wells of a 96-well plate. Following the addition of 200 μL of a 0.15% (*w*/*v*) ninhydrin (Sigma N4876) solution in glacial acetic acid (Carlo Erba Reagents 302031), absorbance was immediately measured using a Ledetect 96 plate reader (Labexim, Lengau, Austria) equipped with an LED plugin at 540 nm, and the plate was incubated for 12 min at 60 °C. The plate was then brought back to RT, and the increase in absorbance at 540 nm was measured 15 to 20 min after ninhydrin addition. P5C content was calculated based on a molar extinction coefficient of 4600 M^−1^ cm^−1^. In the latter case, 100 μL of proper dilutions of the sample was added with the same volume of a 2 mg mL^−1^ solution of *o*AB (Sigma A9628) in ethanol (Carlo Erba Reagents 414605) in a 96-well plate. The colour was allowed to develop at RT, while measuring the absorbance at 1 min intervals with the plate reader equipped with an LED plugin at 444 nm. The reaction was usually completed within 15 min. Parallel blanks were carried out by mixing samples with 100 μL ethanol, to subtract the aspecific absorbance of samples at this wavelength. The P5C content was then calculated on the basis of a molar extinction coefficient for the complex P5C-*o*AB of 2940 M^−1^ cm^−1^.

### 2.4. Plant and Cultured Cell Growth Conditions

Seeds of wild-type and p5cdh knock-out mutant [38] Arabidopsis *thaliana* Heynh., ecotype Col-0, were surface-sterilized for 2 min in ethanol and for 5 min in a 3% NaClO solution containing 0.04% Triton X-100 (Sigma T8787) under vacuum. Following extensive washing with sterile distilled water, seeds were subjected to stratification for 48 h at 4 °C and then allowed to germinate on agarized (7‰) half-strength MS salts [39] (M0245, Duchefa Biochemie, Haarlem, The Netherlands) in Magenta GA7 vessels (6⇨ × 6⇗ × 20⇧ cm). Growth proceeded at 24 ± 1 °C in an FOC 200IL incubator (Velp Scientifica, Usmate Velate, Italy) equipped with 2 lighted shelves (30.000 lux/shelf), each with six LED bars, under an 8 h:16 h day/night photoperiodic cycle. Cell suspension cultures of the same genotypes were grown in MS medium supplemented with 30 g L^−1^ sucrose (Duchefa S0809) and 0.5 mg L^−1^ of both 2,4-dichlorophenoxyacetic acid (Sigma D7289) and 6-benzylaminopurine (Duchefa B0904). Incubation was in dim light at 24 ± 1 °C on a rotary shaker (90 rpm) in 500 mL Erlenmeyer flasks containing 125 mL liquid medium. Cultures were maintained in continuous balanced growth by transferring 25 mL aliquots to 100 mL of fresh medium every week.

### 2.5. Extraction and Quantitation of δ^1^-Pyrroline-5-carboxylic Acid from Plant Tissues

Plant material (about 2 g seedlings or 10 g cultured cells) was harvested, resuspended with 10 or 2 mL g^−1^ of 50 mM HCl, respectively, and extracted with 1 g g^−1^ quartz sand (Aldrich 274739) and a pestle in an ice-cold mortar for 10 min. Following centrifugation at 12,000× *g* for 5 min, the supernatant was loaded onto a Dowex AG50 WX4 (200–400 mesh) column (acid form; 1.5 cm diameter, 5.0 mL bed volume) equilibrated with water, at natural flow. Isocratic elution proceeded with 1 M HCl for the collection of 1.5 mL fractions.

### 2.6. P5C Reductase Heterologous Expression, Purification, and Assay

Rice P5C reductase was expressed in *Escherichia coli*, affinity-purified, and assayed as previously described [37]. The purified enzyme was used to quantify L-P5C by measuring the P5C-dependent oxidation of NADPH. With this aim, 100 μL sample aliquots were placed in wells of a 96-well plate and added with the same volume of a pre-warmed (37 °C) mixture containing 100 mM Tris-HCl buffer, pH 7.5, 0.5 mM NADPH (A328742500 Thermo Fisher, Waltham, MA, USA), and 40 ng of the purified protein, corresponding to about 0.28 nkat. The reaction was allowed to proceed at RT, and the absorbance was measured at 1 min intervals with the plate reader equipped with an LED plug-in at 340 nm. When a plateau was reached, usually within 30 min, the decrease in absorbance was calculated with respect to controls carried out in the absence of P5C. The corresponding consumption of NADPH was quantified based on a molar extinction coefficient of 6220 M^−1^ cm^−1^.

### 2.7. Proline Dehydrogenase Extraction and Assay Methods

Suspension-cultured cells were harvested by vacuum filtration on filter paper, weighed, and resuspended in 2 mL g^−1^ of isotonic buffer (50 Tris-HCl, pH 8.0, containing 400 mM mannitol [Duchefa M0803] and 2 mM EDTA [Sigma E9884]). Cells were extracted with a Teflon-in-glass potter homogenizer with 3 × 10 strokes, and the homogenate was centrifuged for 5 min at 4 °C at 1000× *g*. The pellet was discarded, and the supernatant was further centrifuged for 5 min at 12,000× *g*. The obtained pellets were washed twice with isotonic buffer and finally resuspended in ipotonic buffer (isotonic buffer lacking mannitol). After 30 min on ice, the samples were further homogenated as above and centrifuged for 5 min at 12,000× *g*. The supernatant, showing ProDH activity, was stored at 4 °C and used within a few hours.

Proline dehydrogenase activity was measured at 35 °C following either the reduction of the artificial electron acceptor dichlorophenol indophenol (DCPIP) [7] or the synthesis of the physiological product, P5C. In the former method, a limiting amount of the enzyme was incubated in a final volume of 200 μL in wells of a 96-well plate in the presence of 50 mM Tris-HCl buffer, pH 8.0, 5 mM MgCl_2_, 50 μM FAD (Sigma F6625), 1 mM NaN_3_ (Sigma S2002), 0.4 mM phenazine methosulfate (Sigma P9625), 0.4 mM DCPIP (Sigma D1878), and 100 mM L-proline (Sigma P0380). The assay was started by the addition of the pre-warmed enzyme. At 30 s intervals and up to 10 min, a decrease in absorbance was recorded using the plate reader equipped with a 595 nm plug-in. Activity was calculated by linear regression of data, on the basis of calibration curves obtained under the same conditions with a DCPIP standard. In the latter method, a limiting amount of the enzyme was incubated in the presence of 50 mM Tris-HCl buffer, pH 8.0, 5 mM MgCl_2_, and 50 μM FAD in a final volume of 150 μL. The assay was started by the addition of 100 mM L-proline. At 5 min intervals and up to 30 min, activity was blocked by the addition of 15 μL of a 30% (*w*/*v*) solution of 5-sulfosalicylic acid (Aldrich 247006). Following centrifugation for 1 min at 12,000 × *g*, 100 μL of the deproteinized supernatant was transferred into wells of a 96-well plate and the concentration of P5C was quantified with *o*AB, as described above. Proteins were measured by the Coomassie Brilliant Blue method [40], using bovine serum albumin (Sigma A9647) as the standard.

### 2.8. Statistical Analysis

Linear regression, ANOVA and correlation analysis were performed using Prism 6 for Windows (Version 6.07; GraphPad Software, San Diego, CA, USA).

## 3. Results

### 3.1. Fractionation of Extracts by Cation Exchange Chromatography Followed by the oAB Assay Allowed Detection in Plant Tissues of P5C Levels Exceeding 20 nmol g^−1^

**Figure 2:**
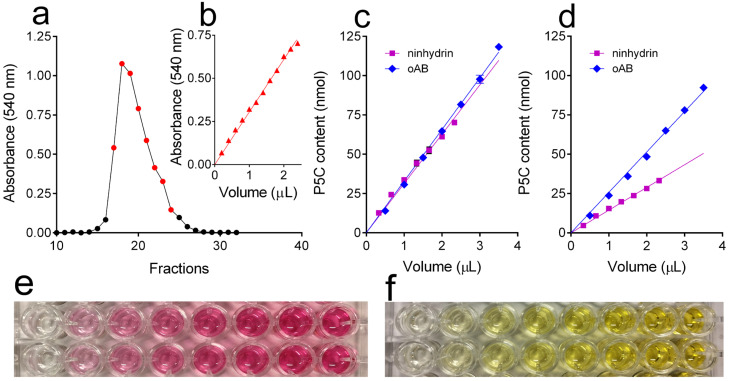
Column purification and quantitation of P5C. (**a**) dl-P5C was synthesized by the periodate oxidation of hydroxylysine, and the reaction mixture was fractionated onto a Dowex AG50 WX4 (200–400 mesh) column, which was eluted with 1 M HCl. P5C content in fractions was measured by the acid ninhydrin assay. Fractions containing more than 5 mM dl-P5C, which were pooled and used for the subsequent experiments, are shown in red. (**b**) The analysis of increasing volumes of pooled fractions allowed a reliable estimation of dl-P5C concentration in the obtained batch of the compound. (**c**) P5C quantitation with the acid ninhydrin and the *o*AB assay method provided almost identical results in freshly prepared solutions, whereas (**d**) different values were found after 1 year of storage at 4 °C in the dark. (**e**) The reaction of increasing amounts of dl-P5C with ninhydrin led to the formation of a cherrish red product with ε = 4600 M^−1^ cm^−1^ at 540 nm, while (**f**) the reaction with *o*AB formed a yellow adduct with ε = 2940 M^−1^ cm^−1^ at 444 nm.

dl-P5C can be chemically synthesized by the metaperiodate oxidation of hydroxylysine and is resolved from ammonia, formaldehyde, and unreacted hydroxylysine by cation-exchange chromatography (Figure 2a). Inseveral batch preparations, the final yield ranged from 67 to 94%. In purified samples, the compound may be detected (Figure 2a) and quantified (Figure 2b) by reaction with either ninhydrin under acidic conditions (Figure 2e) or *o*AB (Figure 2f). The two methods yielded almost identical results when applied to freshly synthesized solutions (Figure 2c; overlapping 95% confidence intervals for slopes: 31.98 to 33.70 and 29.70 to 32.98 for *o*AB and ninhydrin, respectively). However, remarkably different results were evident for aged solutions, with the concentration estimated by the *o*AB method being higher than that estimated by the ninhydrin assay (Figure 2d: non-overlapping 95% confidence intervals for slopes: 24.76 to 26.62 and 13.92 to 14.93 for *o*AB and ninhydrin, respectively). Such a discrepancy depends on the gradual decomposition of P5C, which may only be slowed down by storage at 4 °C in the dark under strongly acidic conditions. *o*AB also reacts with degradation products, leading to an overestimation of P5C concentration [28]. On the contrary, ninhydrin does react only with P5C and also provides reliable results with an aged solution. After 1 year of storage under the above-described conditions, the P5C residual concentration ranged from 50 to 30% of the initial value (Figure 2c,d).

However, both assay methods were found unreliable for measuring P5C levels in crude extracts from plant tissues. In the case of ninhydrin, most free amino acids also reacted, yielding a blue adduct with maximal absorbance at 535–540 nm, largely overlapping with that of the reddish adduct with P5C. From a quantitative point of view, the reaction with amino acids proceeded with much lower efficiency, with a molar extinction coefficient of about 80 M^−1^ cm^−1^. This notwithstanding, with their concentration being one hundredfold higher than that of P5C, their presence masked that of the intermediate. In the case of *o*AB, the ethanol required to solubilize the reagent caused precipitation of proteins in extracts, interfering with the absorbance at 444 nm. Preliminary treatment of extracts with perchloric acid was found to be useful in removing proteins. However, this led to a significant dilution of the samples that hampered the detection of low concentrations of P5C.

**Figure 3:**
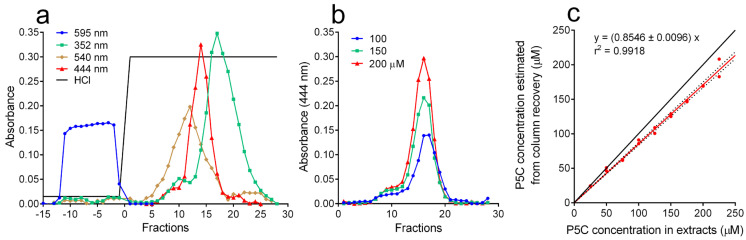
Quantification of P5C in extracts from *A. thaliana* seedlings. (**a**) Seedlings (about 10 g FW) were extracted in 50 mM HCl. Cell-free extracts were spiked with 200 μM dl-P5C and 10 mM l-proline and loaded onto a Dowex AG50 WX4 (200–400 mesh) column that was washed with 50 mM HCl and then eluted with 1 M HCl. Fractions were analyzed for protein (A_595_, Bradford assay [40]) amino acid and proline (A_540_ and A_352_, respectively, acid ninhydrin assay [35]) and P5C (A_444_, *o*AB assay [28]) content. (**b**) The same cell-free extract was added with increasing concentrations of dl-P5C, as indicated, and fractionated by cation exchange chromatography. P5C content in fractions was estimated by the *o*AB assay. (**c**) The analysis of extracts spiked with different dl-P5C concentrations allowed us to calculate recovery from the column, providing a reliable estimation of P5C content in plant tissues. Two replications were carried out for each dose; the identity (black solid) line and the 95% confidence band (dotted lines) are also shown.

To overcome such limitations, a protocol was set up based on extract fractionation by cation-exchange chromatography (Figure 3). Proteins were not retained by the Dowex AG50 WX4 column and could be resolved from P5C, while several amino acids were retained and subsequently eluted under isocratic conditions (Figure 3a). As a consequence, the ninhydrin method could not be used to detect and quantify P5C in the eluate, with its peak being unresolved from those of other amino acids. However, P5C was easily detectable by the *o*AB assay, with the obtained peak being proportional to its concentration (Figure 3b). The analysis of extracts spiked with known concentrations of the compound (Figure 3c) showed a highly significant correlation between P5C concentration in samples and its estimation (*p* < 0.0001 in a Pearson two-tailed test) and allowed us to evaluate the recovery (about 85%) and to estimate the detection limit of the method (about 20–30 nmol g^−1^ FW under the experimental condition used). P5C concentrations in all subsequent experiments were calculated taking into account the recovery value. Interestingly, although the ninhydrin method did not provide reliable results for P5C, it could be used for quantitating proline in the same samples, showing for the proline-ninhydrin adduct a peculiar peak of absorbance at 352 nm (Figure 3a) [35].

### 3.2. P5C Levels in Plant Tissues and Cultured Cells Were Found to Be Below the Detection Limit of the Method but Could Be Measured in Proline-Treated Seedlings of an A. thaliana p5cdh Mutant

**Figure 4:**
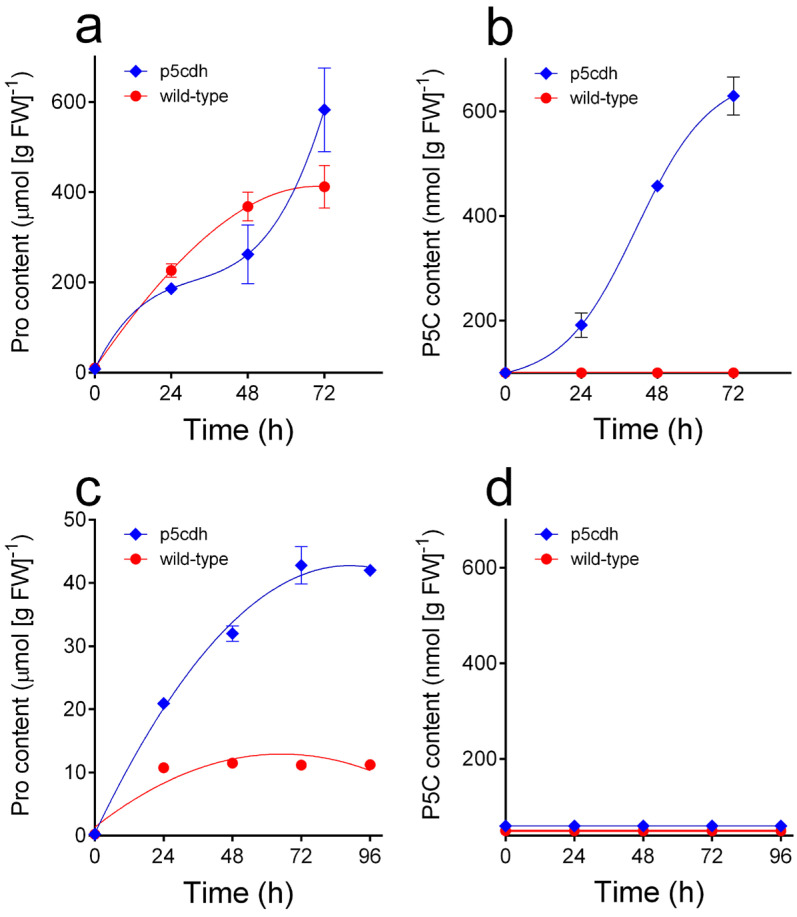
P5C accumulation in proline-treated plants and cultured cells of wild-type and p5cdh *A. thaliana*. Three-week-old Arabidopsis seedlings were treated with a 1 M sterile solution of proline that was added on top of the agarized medium so as to obtain a final concentration of 50 mM. Cell suspension cultures were similarly treated with 25 mM proline, added as a 2 M sterile solution. At increasing time after the addition, plants (about 1.2 g FW) and cultured cells (about 10 g) were extracted in 50 mM HCl, and extracts were fractionated by cation-exchange chromatography. Proline and P5C were quantitated from the recovery in the eluate from the Dowex AG50 WX4 (200–400 mesh) column, as shown in Figure 3. Three replications were performed for each treatment, and mean values ± SE are reported. (**a**) Proline content in seedlings; (**b**) P5C content in seedlings; (**c**) proline content in cultured cells; (**d**) P5C content in cultured cells. In panels b and d, the *y*-axis starts at the detection limit for P5C under the experimental conditions used for seedlings and cultured cells, respectively.

When the protocol was applied to extracts from untreated seedlings and suspension-cultured cells of *A. thaliana*, no P5C was detectable in the eluate from the Dowex AG50 WX4 column. The same results were also obtained in the case of the Col-0 insertional mutant lacking a functional P5CDH [38]. Different results were on the contrary obtained with proline-treated seedlings (Figure 4). The addition to the culture medium of 50 mM proline caused a rapid uptake and accumulation of the amino acid in plant tissues, without significant differences between genotypes (*p* = 0.8603 in a two-way ANOVA; Figure 4a). In wild-type seedlings, P5C levels remained below the detection limit, whereas in p5cdh seedlings, proline accumulation was concomitant with a progressive increase in P5C content that approached millimolar concentrations (Figure 4b). Interestingly, when similar experiments were performed with cell suspension cultures, the treatment with 25 mM exogenous proline caused a rapid increase in intracellular content in wild-type cells to a level (about 10 μmol g^−1^) that was maintained thereafter. In p5cdh cells, the intracellular concentration of proline was raised to 4-fold higher levels (*p* < 0.0001 in a two-way ANOVA) and reached a plateau only after a further 48 h (Figure 4c). However, in neither case did this cause P5C accumulation above the detection limit (Figure 4d).

### 3.3. The Protocol Allowed Measurement of the Incorporation of Exogenous P5C by Suspension Cultures, Which Was Similar in A. thaliana Wild-Type and p5cdh Cells

**Figure 5:**
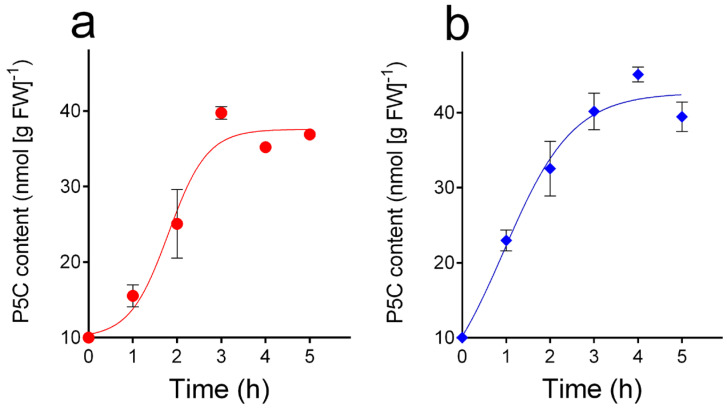
P5C levels in *A. thaliana* cultured cells following the addition of the compound to the culture medium. Cell suspension cultures were treated with 200 μM dl-P5C. After an increasing period of time after the addition, cells were harvested, washed, and extracted in 50 mM HCl. P5C was purified from cell free extracts by cation exchange chromatography and quantified as described in Figure 3. Two independent experiments were performed, and data were combined; means ± SD are reported. The *y*-axis starts at the detection limit for P5C under the experimental conditions used. (**a**) Wild-type cells; (**b**) p5cdh cells.

When cultured cells were treated with exogenous dl-P5C, low but detectable intracellular levels of the compound were found at increasing time after the addition (Figure 5). In wild-type cells, the concentration of the intermediate reached a maximal level of about 40 nmol g^−1^ (FW) 3 h after the treatment and did not increase thereafter despite the higher concentration (200 nmol mL^−1^) in the culture medium (Figure 5a). A similar pattern was obtained with the *A. thaliana* p5cdh mutant (Figure 5b), with the only difference being a significantly more rapid initial increase (*p* = 0.002 in a two-way ANOVA).

### 3.4. Following Sample Deproteinization with Sulphosalycilic Acid, the oAB Assay Was Found Useful to Assay ProDH, Providing More Reliable Results than the DCPIP Reduction Assay Method

**Figure 6:**
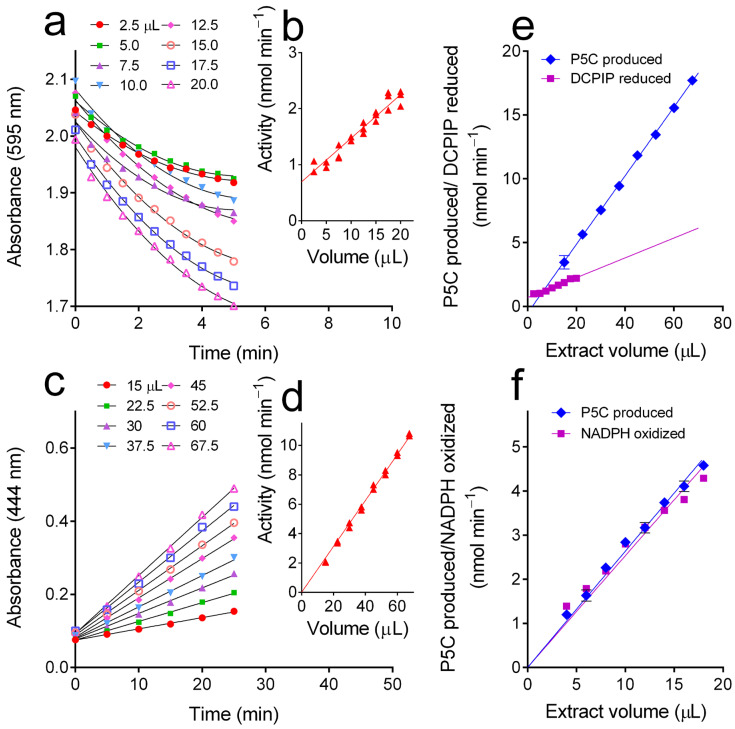
Assay of ProDH activity in partially purified extracts from an *A. thaliana* p5cdh mutant. Enzyme activity was assayed by either (**a**) the conventional assay method based on DCPIP reduction or (**c**) the *o*AB method measuring P5C production following sample deproteinization with 3% (*w*/*v*) 5-sulphosalicylic acid. Insets show the quantitative relationship between sample volume and the activity value obtained, as well as the experimental variability ((**b**,**d**) for the DCPIP and the *o*AB method, respectively). (**e**) Comparison of the mean values obtained with the same extract by means of the two methods, showing a significant underestimation of activity values by the DCPIP assay. (**f**) Comparison of the results obtained when P5C production was followed in the same extracts by means of either the *o*AB method or a coupled assay method in which the P5C-dependent NADPH oxidation by P5CR was measured.

The *o*AB method was also used to measure ProDH activity, which is usually assayed by a protocol that follows the proline-dependent reduction of an artificial electron acceptor, DCPIP [7]. Extracts from cultured cells of the *A. thaliana* p5cdh mutant were used to rule out the possibility that any further oxidation of P5C may occur in the reaction mixture. Preliminary experiments yielded unreliable results due to the precipitation of proteins when the alcoholic solution of *o*AB was added to the enzymatic reaction mixture. The drawback was overcome by precipitating proteins through the addition of 3% (*w*/*v*) 5-sulphosalicylic acid, which did not interfere with the subsequent colorimetric reaction. Following centrifugation, pellets were discarded and P5C in the supernatant could be measured. A comparison of the results obtained with either method on the same enzyme preparations is reported in Figure 6. With the DCPIP assay, reaction kinetics lost linearity with time within a few minutes (Figure 6a). Moreover, a high variability was found among replicates, and the presence of aspecific DCPIP reduction was also evident (Figure 5b). With the *o*AB assay, on the contrary, activity was linear with time for at least 25 min (Figure 6c). Variability among samples was lower, and an exact correspondence was found between the extract volume used and the amount of P5C produced (Figure 6d). Remarkably, the activity rates obtained in the two cases were significantly different (non-overlapping 95% confidence intervals for slopes: 0.2617 to 0.2762 and 0.06580 to 0.08962 for P5C produced and DCPIP reduced, respectively), with those measured as DCPIP reduction being much lower than those obtained measuring P5C production (Figure 6e).

To shed some further light on this discrepancy, ProDH was also assayed by means of a third, discontinuous method in which P5C production was coupled with NADPH oxidation by purified recombinant P5CR [37]. The results were in this case not statistically different from those obtained with the *o*AB method (*p* = 0.1102 in a two-way ANOVA; Figure 6f), strengthening the reliability of the latter.

## 4. Discussion

Many studies in recent decades have pointed to P5C as a key intermediate in both proline synthesis and catabolism, whose metabolic fate seems able to lead the plant cell to strikingly different responses, such as PCD or stress adaptation [11,12,17]. However, despite numerous efforts, several aspects still await elucidation. For instance, it is not clear whether P5C itself is toxic for the cell or whether its effect is mediated by other signal transduction mechanisms. The existence of a P5C transporter able to mediate its efflux from the mitochondrion to the cytosol, hypothesized as an essential requirement of the proline–P5C cycle, has not been demonstrated, yet [11]. Recent data also suggested the possibility that during proline catabolism P5C is not released by ProDH but is converted directly to glutamate or ornithine through substrate channelling between ProDH and P5CDH or OAT [27]. However, this would be inconsistent with the occurrence of the proline–P5C cycle [9], and it is not clear whether substrate channelling is a constitutive mechanism or is induced under some specific conditions.

A drawback that limits the possibility of shedding some further light on these aspects is the difficulty in detecting and quantifying P5C in extracts from plant tissues. The experimental protocol herein set up was found useful for concentrating P5C and removing most interfering substances, allowing its measurement by means of the *o*AB assay without the need for sophisticated equipment. The ninhydrin assay, on the contrary, provided unreliable results due to the presence in plant extracts of high concentrations of free amino acids able to react under the same conditions (Table 1). The analysis of spiked extracts showed that concentrations as low as 30–40 nmol g^−1^ can be detected in this way. This notwithstanding, the analysis of extracts from seedlings or cultured cells of *A. thaliana*, treated or not with exogenous proline to induce ProDH expression, failed to detect the presence of the intermediate. This would be consistent with the occurrence of substrate channelling between ProDH and P5CDH, also taking into account that a relatively high K_M_ for P5C has been found for P5CDH (about 200–400 μM with either NAD^+^ or NADP^+^ [8,36]). However, the analysis of seedlings of a p5cdh mutant, in which the P5C produced by ProDH cannot be further oxidized to glutamate, showed that treatment with exogenous proline leads to the accumulation of intermediate-to-high levels, two orders of magnitude over the detection limit of the method. Interestingly, detectable amounts were found in seedlings but not in proline-treated suspension-cultured cells of the p5cdh mutant. Several factors could explain such apparent inconsistency. The lack of P5C accumulation in cultured cells could depend on much lower levels of ProDH activity. Previous literature data indeed show that *ProDH2* expression is reduced by treatment with exogenous sucrose and almost completely abolished in cultured cells grown heterotrophically (Figure 3 and Figure 4 in [41]). A similar effect was found for *ProDH1* in sucrose-treated seedlings (Figure 4 in [41]). However, the concomitant treatment with 20 mM proline almost completely reverted the repression of both isogenes (Figure 4 in [41]). Moreover, high levels of ProDH1 mRNA were found in cultured cells, similar to those in flowers and much higher than those in seedlings. It is therefore unlikely that the striking difference found in P5C accumulation could be the consequence of an almost complete lack of ProDH expression in cultured cells. However, the intracellular levels of proline found after increasing time had passed following treatment were much higher in seedlings than in plants. As a consequence, also, the rates of ProDH activity, which has been shown to have a high K_M_ for proline (31 mM [7]), could be much lower in cultured cells despite similar expression. Moreover, actively proliferating cells are likely to express high levels of P5CR, which is required to produce proline needed for protein synthesis. Consequently, the P5C released by proline oxidation could be transferred into the cytosol and reduced back to proline by P5CR. If so, this could represent a result supporting the existence of a P5C transporter in the mitochondrial membrane. In any case, further experiments will be required to discriminate among these possibilities.

The protocol was also found suitable to follow the intracellular level of the intermediate in suspension-cultured cells after an increasing period of time after treatment with exogenous P5C. Low but detectable and increasing amounts were evident following the addition, but a plateau was rapidly reached, and the internal level did not further increase thereafter. Very similar results were obtained for wild-type and p5cdh cultures. These data strengthen the possibility that in actively proliferating cells, P5C is rapidly used in the cytosol by P5CR, yielding the amounts of proline needed for protein synthesis.

The *o*AB assay was also adapted to measure the activity of ProDH. This enzyme, functionally located in the inner mitochondrial membrane, is believed to transfer electrons directly to the respiratory chain [7]. As a consequence, its activity cannot be measured following NAD(P)^+^ reduction, as is the case for most oxidoreductases [42]. Therefore, ProDH is commonly assayed by following the reduction of an artificial electron acceptor, DCPIP. This method has some advantages (Table 2), such as a relatively high molar extinction coefficient (about 20,000 M^−1^ cm^−1^, depending on the pH) and the fact that the consequent decrease in absorbance at 595 nm can be monitored continuously. However, activity is not perfectly linear with time, and DCPIP could be at least in part re-oxidized by other mitochondrial enzymes, or part of the proline-derived electrons could be transferred in any case to the respiratory chain. This is the reason why the reaction mixture is routinely added with inhibitors of the latter, such as sodium azide and cyanide [7,42]. The *o*AB assay has a lower molar extinction coefficient (2940 M^−1^ cm^−1^), thereby requiring the incubation of higher extract volumes. Moreover, it is a discontinuous method in which the enzymatic reaction has to be stopped to proceed to sample deproteinization before the colorimetric reaction with P5C can be carried out. This notwithstanding, several advantages are evident. The physiological reaction is measured. Activity is linear with time for at least 25 min. The presence in the same extract of P5CDH and/or P5CR would not interfere with the assay, since further P5C metabolization by these enzymes requires the presence in the assay mixture of NAD(P)^+^ [36] and NAD(P)H [37], respectively. Interestingly, when the two methods were herein used to quantify ProDH levels in the same extracts, activity values obtained with the *o*AB assay were more than twice those obtained with the DCPIP assay. The reliability of the results of the *o*AB method was strengthened by the use of a third assay method, in which the P5C released by ProDH is indirectly measured as the P5C-dependent consumption of NADPH by recombinant P5CR. This method cannot be used in partially purified preparations, since the NADP^+^ produced by P5CR could be reduced back in the presence of P5C by P5CDH. To avoid this drawback, we used extracts from cultured cells of the *A. thaliana* p5cdh mutant. The results obtained were almost perfectly overlapping those of the *o*AB assay. Data thus suggest that the DCPIP method could significantly underestimate ProDH activity and point to the use of the *o*AB assay as a reference method for biochemical studies on this enzyme.

## 5. Conclusions

A simple and easily affordable protocol to extract, concentrate, and quantify l-P5C from plant tissues was set up and validated. The method was found to be able to measure P5C uptake in *A. thaliana* cultured cells, as well as demonstrating P5C accumulation in proline-treated plants of a p5cdh mutant. Following sample deproteinization, the colorimetric assay with *o*AB also allowed a reliable measurement of the physiological reaction of ProDH, partially purified from cultured cells.

## Figures and Tables

**Table 1:** Comparison between the two colorimetric methods available for P5C detection.

	Ninhydrin Assay	*o*AB Assay
molar extinction coefficient	4600 M^−1^ cm^−1^ at 540 nm	2940 M^−1^ cm^−1^ at 444 nm
sensitivity ^1^	about 670 μM	about 75 μM
specificity	low	high
optimal solvent for cell extraction	3% (*w*/*v*) sulfosalicylic acid	50 mM HCl
solvent required for the assay	glacial acetic acid	ethanol
assay temperature	50 °C	room temperature
time for colour development	20 min	15 min
possible interferences	most amino acids react under the same conditions, even if to a lesser extent	proteins may precipitate due to the presence of 50% ethanol in the reaction mixture
pre-requisites	resolution from all other free amino acids	sample deproteinization

^1^ Concentration of P5C that needs to be present in a sample to give 0.1 optical units under the experimental conditions described in Materials and Methods.

**Table 2:** Comparison between the protocol herein described and the two other main methods to measure ProDH activity.

	P5C Assay	DCPIP Assay	NAD(P)H Assay
molar extinction coefficient	2940 M^−1^ cm^−1^ at 444 nm	about 20,000 M^−1^ cm^−1^ at 595 nm, depending on pH	6220 M^−1^ cm^−1^ at 340 nm
reaction measured	direct	direct	coupled: need the addition of purified P5CR (or P5CDH ^1^)
type of assay	discontinuous; requires sample deproteinization before *o*AB addition	continuous	discontinuous; requires absence or heat inactivation of endogenous P5CDH before the addition of P5CR and NADPH
reaction measured	physiological	artificial	physiological
linearity with time	good	poor	good
sensitivity	lower	higher	lower
time required for the assay	from 30 to 60 min	from 10 to 20 min	from 45 to 90 min
possible interferences	none in desalted extracts	presence of endogenous dehydrogenases able to re-oxidate DCPIP; partial transfer of electrons from ProDH to the respiratory chain	none in desalted extracts
pre-requisites	desalting step to remove NAD(P)(H), whose presence could allow endogenous P5CR/P5CDH to further metabolize the P5C produced by ProDH	complete inhibition of dehydrogenases able to use reduced DCPIP as an electron donor; complete inhibition of electron transfer from ProDH to the respiratory chain	availability of purified P5CR/P5CDH; desalting step to remove NAD(P)(H), whose presence could allow endogenous P5CR/P5CDH to further metabolize the P5C produced by ProDH

^1^ The coupled assay may be performed also by following the increase in absorbance at 340 nm due to the P5C-dependent NAD^+^ reduction by exogenous P5CDH.

## Data Availability

Raw data will be made available by the corresponding author upon reasonable request.

## Abbreviations

The following abbreviations are used in this manuscript:

oAB	ortho-aminobenzaldehyde
DCPIP	dichlorophenol indophenol
GSA	glutamate γ-semialdehyde
OAT	ornithine-δ-aminotransferase
P5C	δ1-pyrroline-5-carboxylic acid
P5CDH	δ1-pyrroline-5-carboxylate dehydrogenase
P5CR	δ1-pyrroline-5-carboxylate reductase
P5CS	δ1-pyrroline-5-carboxylate synthetase
PCD	programmed cell death
ProDH	proline dehydrogenase
